# Factors Associated with Adolescents’ Self-Efficacy to Meet with their Health Care Provider Alone

**DOI:** 10.1177/10901981251357341

**Published:** 2025-08-22

**Authors:** Albert C. Hergenroeder, Blanca Sanchez-Fournier, Cassandra Enzler, Mary Majumder, Beth H. Garland, Constance M. Wiemann

**Affiliations:** 1 Texas Children’s Hospital, Baylor College of Medicine, Houston, TX, USA; 2 Vital Research, Los Angeles, CA, USA; 3 Baylor College of Medicine, Houston, TX, USA

**Keywords:** self-efficacy, theories, adolescents, population groups, young adults, behavioral theories, health behavior, general terms

## Abstract

Adolescents with special health care needs (ASHCNs) must develop self-management skills to effectively transition into adult-based care. This requires having the self-efficacy to meet with their health care provider independent of caregivers. This study aims to identify the factors associated with self-efficacy in meeting with a provider alone among ASHCN preparing for this transition.

Eighty-three English-speaking 18-year olds with renal, gastrointestinal, neurologic, or rheumatologic diseases, and their English- or Spanish-speaking caregivers were recruited at a large children’s hospital, and completed a one-time assessment as the historical control group for a larger intervention study. The main outcome measure was self-efficacy to meet with their health care provider alone. Factors evaluated for their independent association with self-efficacy using linear regression included Self-Determination Theory constructs (autonomy, competence, and relatedness); importance of meeting with their provider alone; and whether they had met with their provider alone in the past 12 months.

Seventy-percent of ASHCN had met alone with their provider in the last 12 months. Female gender, perceived competence, perceived provider support for autonomy, and having met with their provider alone in the last 12 months were associated with self-efficacy in meeting with their provider alone.

ASHCNs who feel competent in managing their health and who perceive their providers as supporting their self-management autonomy also feel the most efficacious in meeting with their provider alone. Pediatric providers can help prepare ASHCN for transition by meeting with them alone.

Adolescents with special health care needs (ASHCN) who are preparing to successfully transition to adult-based care must develop the self-management skills required to flourish in the adult health care system (Lozano & Houtrow, 2018). During adolescence, when the management of a chronic condition ideally shifts from caregivers to the ASHCN, detrimental behaviors such as poor adherence can emerge leading to adverse health outcomes (Lerch & Thrane, 2019). Alternatively, if self-management support from caregivers and health care providers is provided during adolescence as the caregiver-ASHCN and provider-ASHCN relationships are evolving, then improved outcomes can result (Lozano & Houtrow, 2018). This support can be manifest as the caregiver allowing, and the pediatric provider setting the expectation that the ASHCN spend time with the pediatric provider alone during clinic visits. For example, having an independent, positive relationship with the provider and medical team promoted a sense of competence and belief in patients’ ability to master a demanding compliance regime in patients with cystic fibrosis (Lerch & Thrane, 2019; Sawicki, Heller, et al., 2015). Yet the caregivers of only 56% of 12- to 17-year olds with special health care needs reported their ASHCN having time alone with their health care provider at their last preventive care visit (Child and Adolescent Health Meaurement Initiative, 2021–2022).

The American Academy of Pediatrics recommends that to promote increased independence around health care decision-making, all adolescents and young adults should be given the opportunity to meet with providers without parents/caregivers present (Alderman & Breuner, 2019). Adolescents, including those with ASHCN, who are not able to meet with their pediatric providers by themselves miss opportunities to develop confidence in communicating with future adult health care providers. Adolescents who are less reluctant to communicate with providers about their disease and symptoms may become more self-reliant in adherence with self-management activities (Kayle et al., 2016; Lerch & Thrane, 2019). The opportunity to communicate about their disease could be enhanced by having time alone with their pediatric provider to prepare for transitioning to adult care. Literature on factors associated with improved self-management of ASHCN has focused on individual, family, and community domains (Camp-Spivey et al., 2022) and caregiver-adolescent relationships. Despite evidence that time alone with their provider is a core element in health care transition (HCT) planning, requiring the caregiver to be invited out of the room during a clinic visit, this practice is not consistently described as an important activity in promoting self-management (Lebrun-Harris et al., 2018).

Previous research has shown that motivation is essential for a person to adopt any specific behavior (Ryan & Deci, 2002). Self-determination theory (SDT) is an empirically based theory of human motivation that has proven useful in promoting long-term health behaviors (Deci & Ryan, 1985; Ryan & Deci, 2000). According to SDT, internal motivation and behavioral performance stem from three fundamental psychological needs: competence, autonomy, and relatedness (Deci & Ryan, 2012). These elements can be operationalized to address the challenges ASHCN encounter in attaining self-management of their health: competence (the necessary skills and confidence to engage in specific behaviors), autonomy (the ability to act in accordance with one’s own values and desires), and relatedness (the support for autonomy from provided by one’s social network, including family and health care professionals (Stephens et al., 2021). Relatedness, as defined by autonomy support, is developmentally appropriate given the skills being fostered during this life period (e.g., independent self-management skills). This article focuses on self-efficacy of the ASHCN in meeting alone with their provider and, consistent with the SDT framework, explores possible associations between provider and caregiver behaviors and ASHCN self-efficacy via measures of perceived autonomy support.

This study is part of a larger evaluation of an intervention targeting ASHCN, their caregivers, and providers designed to maximize opportunities for ASHCN to develop competence and autonomy as they prepare for HCT to adult-based care. In this study, we identified factors influencing self-efficacy in meeting with a provider independently among ASHCN preparing for this transition, using an SDT framework. We hypothesized that the three SDT constructs—competence, autonomy, and autonomy support/relatedness—would have a positive e relationship with ASHCN’s self-reported self-efficacy to meet with their provider alone.

## Methods

### Study Design

#### Participants

Eighteen-year olds were recruited from the Renal, Gastroenterology, Neurology, and Rheumatology Services at a large children’s hospital in the southwest United States to participate in the completion of a one-time assessment to better understand their preparation for HCT and their experience of preparing for that transition. One caregiver (typically a parent) of each participant also responded to a short questionnaire. These 18-year old and caregiver dyads also served as the historical control group for a larger intervention study completed at a later date. The study protocol was approved by the Baylor College of Medicine Institutional Review Board for Human Subjects Research.

#### Recruitment

Recruitment occurred between August and December 2020 using three steps: (a) Project staff created lists of potential participants from participating services and submitted them to clinic liaisons for approval; (b) A recruitment letter in English or Spanish and study flyer were mailed to potential participants; and (c) Project staff contacted potential participants after mailing the recruitment letter to explain the study and answer questions.

All genders and racial/ethnic groups were eligible to participate. Eligible ASHCN participants could read and speak English; eligible caregivers could read and speak English or Spanish. ASHCN who could not participate due to specific conditions (e.g., blindness, cognitive impairment, moderate or severe developmental or intellectual delay) were excluded. ASHCN and caregivers provided written consent and agreed to complete a one-time assessment, and were compensated $25.

#### Data Collection and Measures

Assessments took about 30 minutes to complete and were administered prior to scheduled clinic appointments using REDCap (Research Electronic Data Capture) (REDCap Version 8.10.2 2019) accessed through participants’ mobile phones. The assessments obtained socio-demographic information, such as age, self-reported race and ethnicity, gender, and insurance and school status. The SDT constructs described below were also assessed. Given the health disparities that minority ASHCN face in HCT planning (Armstrong et al., 2008; Blum et al., 2002; Stivers & Majid, 2007); this study did not exclude potential participants by race and/or ethnicity.

##### SDT Guided Selection of Factors Associated with Self-Efficacy to Meet With Their Provider Alone

Measures assessing SDT constructs used a version modified for prior studies of ASHCN (Enzler et al., 2021; Stephens et al., 2021) to measure health care management. Following procedures specifically established for these constructs (Levesque et al., 2007; Seiffge-Krenke, 2001), the terms “health” or “health care” or “health care providers” were inserted in each item. For example, “The reason I would [manage my health] is . . .” Or, “I feel that my [health care providers] have provided me with choices and options about how to [manage my health care].” Cronbach alpha coefficients were generated.

***Perceived self-efficacy of the ASHCN to meet with their provider alone*** was assessed using a validated measure of self-efficacy to talk with a provider (van Staa et al., 2011) modified to include terms that are more common in the United States. The measure consists of seven items measured on a 6-point scale from “Not at all confident” to “Very confident” (Cronbach alpha = .93). An example item includes “I could ask my health care provider to explain things until I understand it.” Items are summed and averaged with higher scores indicating higher perceived self-efficacy that the ASHCN could meet with their provider alone.

***Perceived competence*** was measured using the 13-item patient activation measure (PAM, Insignia Health, (Hibbard et al., 2004). Items (strongly agree to strongly disagree) assessed knowledge, skill, and confidence in managing one’s health and health care (Cronbach alpha = .88) and scores range between 0 and 100. The higher the score, the greater the perceived competence. This measure was completed by the ASHCN. An example question is: “When all is said and done I am the person who is responsible for taking care of my health.”

***Health care autonomy*** was measured using the Treatment Self-Regulation Questionnaire (TSRQ) (Levesque et al., 2007). The TSRQ is a 15-item instrument that uses a 7-point Likert-type scale, where 1 indicates “not at all true” and 7 signifies “very true.” There are three subscales: autonomous motivation (6 items, Cronbach’s alpha = .88), controlled motivation (6 items, Cronbach’s alpha = .84), and amotivation (3 items, Cronbach’s alpha = .61). Adolescents scoring high on autonomous motivation are those more motivated to engage in healthy behaviors. An example question is: “The reason I would manage my health care is: because I personally believe it is the best thing for my health.” Adolescents scoring high on controlled motivation are those motivated to please others or avoid disappointment or consequences. An example question is: “The reason I would manage my health care is because others would be upset with me if I did not.” Adolescent scoring high on amotivation are those not motivated by either themselves or others to engage in healthy behaviors. An example question is: “I really don’t think about managing my care.” Due to a low Cronbach alpha, the amotivation subscale was not analyzed for this study.

***Perceived support for health care autonomy (relatedness)*** was measured using the Health Care Climate Questionnaire (HCCQ) (Williams et al., 1996, 1998), yielding two measures of support: a health care provider measure for (Cronbach alpha = .91) and a caregiver/parent measure (Cronbach alpha = .92). Each measure contained six items that ranged from “Very True” (coded 6) to “Not at all true” (coded 0). Items for each scale were averaged. A sample Provider/Parent Autonomy Support item is: “My health care providers/parent convey confidence in my ability to make changes regarding the ways in which I manage my health care.”

***Meeting with provider alone*** was measured using modified questions from the ADAPT Survey (Sawicki, Garvey, et al., 2015) by asking both the ASHCN and caregiver two assessment questions: “In the last 12 months, prior to today’s visit, did you/your adolescent talk with your/their health care provider without you/your parent/guardian in the room? Yes/No.” And “Did you/your adolescent meet with the provider alone at any point during today’s visit? Yes/No.” The caregiver answer was reported and used in subsequent analyses.

ASHCN and caregivers were asked to rate their perceived ***importance of meeting with the provider alone*,** measured on scale of 1 (*Not at all important*) to 10 (*Very important*). The ASHCN rating was used in subsequent analyses.

### Data Analysis

Data were analyzed using the SPSS program Version 25. Preliminary analyses included descriptive statistics for study variables and comparisons of mean self-efficacy scores across the sample, stratified by gender, race and ethnicity, insurance status, and prior visits with provider alone in the past 12 months, including today’s visit. Correlational analyses were also conducted. A criterion of *p* ≤0.20 was applied to identify variables for inclusion in multivariate analyses. Multiple linear regression was employed to assess the independent relationships between the dependent variable—self-efficacy to meet with the provider alone—and the SDT constructs and related factors. Potential covariates were included in Step 1, with subsequent variables analyzed using stepwise regression.

## Results

Eighty-three 18-year olds with renal (*n* = 19), gastrointestinal (*n* = 20), neurologic (*n* = 22), or rheumatologic (*n* = 22) disease participated in the study; their demographic characteristics are described in Table 1. Only five of the ASHCN who were approached about the study refused participation (5/88 = 5.7%).

**Table 1.** Demographic Characteristics (N = 83).

Characteristic	*N* (%)
Age, mean ± *SD*	18.4 ± 0.3
Gender	
Female	45 (54.2)
Male	38 (45.8)
Race^a^	
African-American/Black	11 (13.3)
Caucasian/White	72 (86.7)
Other (American Indian, Middle Eastern)	2 (2.4)
Ethnicity, Hispanic	41 (49.4)
Current student, yes	66 (79.5)
Insurance	
Public/no insurance	41 (49.4)
Private insurance	42 (50.6)

a Numbers for Race sum to more than 83 due to participants who recorded more than one race or ethnicity.

A total of 42 of 83 (51%) caregivers reported that their ASHCN had met with their providers alone in the last 12 months; 58 of 83 (70%) caregivers reported that their ASHCN had met with their provider alone in the last 12 months including today’s visit. Thirty of 83 (37%) caregivers reported that their ASHCN met with their provider alone at today’s visit. Caregivers as compared to their adolescents reported a slightly higher mean perceived importance of the ASHCN meeting with their provider alone (6.5 ± 3.1 vs. 5.8 ± 2.8, *p* = .09). Overall, 21 (25.3%) of ASHCN and 17 (20.5%) of caregivers ascribed low (less than somewhat important) importance to having the ASHCN meeting with their provider alone.

Higher self-efficacy to meet with provider alone was reported by females as compared to males (5.1 ± 0.8 vs. 4.6 ± 1.1, *p* = .03, Table 2) and by ASHCN who had versus had not met with their provider alone in the past 12 months per the Caregiver report (5.0 ± 0.9 vs. 4.5 ± 1.1, *p* = .03). There were no significant (*p* > .20) differences in self-efficacy to meet with provider alone among the sample grouped by race, ethnicity, clinical service, or insurance status.

**Table 2.** Mean Self-Efficacy to Meet With Provider Alone for the Sample Stratified by Gender and Having met With Their Provider Alone in the Last 12 Months.

Gender and met with provider alone	Mean ± *SD*	*p* value
Gender		0.03
Female	5.1 ± 0.8	
Male	4.6 ± 1.1	
Met with provider alone, past 12 months (caregiver report)		0.03
Yes	5.0 ± 0.9	
No/not sure	4.5 ± 1.1	

There were moderate correlations between perceived self-efficacy to meet with provider alone and the SDT constructs perceived competence, provider and parent autonomy support, and autonomous autonomy (Table 3). Controlled autonomy and perceived importance of meeting with provider alone were not correlated with self-efficacy.

**Table 3.** Correlations Between Self-Efficacy to Meet With Provider Alone and Other SDT Constructs.

STD constructs	Correlation coefficient
Perceived competence	0.481*
Provider autonomy support	0.449*
Parent autonomy support	0.413*
Autonomous autonomy	0.368*
Controlled autonomy	–0.05**
Perceived importance of meeting with provider alone	–.058**

* *p* < .01 (two-tailed); **p > .20.

Four factors in the final regression model were significantly associated with the ASHCN self-efficacy in meeting with provider alone (*R*-square = .384, *F* = 12.701 (4, 77), *p* < .001) (Table 4). These were being female (*p* = .023), perceived competence (*p* = .004), provider support for autonomy (*p* = .003), and having met with the provider alone in the last 12 months (*p* = .038). The multivariate model did not retain autonomous autonomy and parent support for autonomy.

**Table 4.** Regression Model Predicting Self-Efficacy to Meet With Provider Alone.

	*R*	*R* square	*F* change	*p* value
Step 1^a^	.237	.056	4.81	.031
Step 2^b^	.535	.286	25.74	<0.001
Step 3^c^	.591	.349	7.64	0.007
Step 4^d^	.620	.384	4.44	0.038
	Final model statistics			
Study construct	Beta	Standardized beta	*t*	*p* value
Gender	.408	.211	2.33	.023
Competence	.020	.303	2.93	.004
Provider autonomy support	.270	.317	3.07	.003
Met with provider alone last 12 months	.406	.193	2.11	.038

a Step 1 gender was entered first as a control variable.

b Step 2 gender, competence.

c Step 3 gender, competence, provider autonomy support.

d Step 4 gender, competence, provider autonomy support, met with provider alone last 12 months (caregiver report).

## Discussion

Using an SDT framework, we hypothesized that the SDT constructs of competence, autonomy, and autonomy support (relatedness) from providers and caregivers would be associated with ASHCN self-efficacy to meet with the provider alone. Results of the linear regression analysis revealed that ASHCN who feel competent in managing their health, those who perceive their providers as supporting their autonomy in managing their health, and those who had experienced meeting alone with their provider also feel the most efficacious to meet with their provider alone. Competence, provider autonomy support, and self-efficacy to meet with the provider alone were consistent with the hypothesis proposed in this study. Previous work by the authors of this article showed that perceived competence and provider autonomy support were positively associated with HCT readiness as measured by the Transition Readiness Assessment Questionnaire (TRAQ) (Stephens et al., 2021). As the TRAQ asks about the frequency of behaviors related to taking responsibility for interactions with health care providers, there is a likely overlap between HCT readiness and self-efficacy in meeting with their provider alone. Given the expectation in adult care that ASHCN be able to meet and converse with their adult providers by themselves, the onus is on pediatric providers to help prepare ASHCN by practicing this behavior while still in the pediatrician’s office. For example, setting the expectation that parents leave the clinic room starting at age 14 (White & Cooley, 2018) and executing this expectation will allow ASHCN to interact with providers alone.

The influential Donabedian model of quality offers insights into understanding elements of effective support for ASHCN’s developing self-efficacy to meet with their pediatric provider alone as a component of high-quality HCT planning (Donabedian, 1966). The Donabedian model directs attention to three components that shape quality of care: the structure of care (i.e., setting and instrumentalities, including provider attributes), the actual process of care, and the outcomes of care. In the context of this study, the first component of care provision, that is, structure, includes the provider attributes of knowing when and how to skillfully invite the caregiver out of the room, emphazing the importance and purpose of meeting alone, and making this a standard activity in the pediatric practice. The second component is the process outcome of actually meeting with the provider alone. In the 2022 national survey for those 12- to 17-year-old ASHCN who had seen a doctor, nurse or other health care provider in the past 12 months, 56% of caregivers reported that their ASHCN had a chance to to speak with a doctor or other health care provider privately (Child and Adolescent Health Meaurement Initiative, 2021–2022). In this study 51% of subjects had met with their provider alone in the prior 12 months, and this increased to 70% if today’s visit, when the survey was administered, was included. One explanation for the higher percentage compared to the national survery is the subjects in this study were 18-year olds compared to 12- to 17-year olds in the national survey, an age group less likely to spend time alone with the provider. In addition, the measure used in this study evaluated meeting with the provider alone over the past year including today’s visit, as compared to their last medical visit as measured in the national survey.

In this study, 25% of ASHCN and 21% of caregivers did not think meeting alone with the provider was important. This is not surprising considering 30% had not done so, allowing for the ASHCN and caregivers’ conclusions that if it was important, it would have already occurred. Enabling the ASHCN to meet alone with the provider to develop relationships independent of their caregiver underscores the importance of knowing how to talk to providers on their own, which is expected in adult-based care. It also demonstrates provider support for the ASHCN’s evolving autonomy and competence, ideally resulting in improved self-efficacy to meet alone with future providers.

ASHCN competence, as measured by the PAM, was significantly related to self-efficacy in meeting with the provider alone. One-on-one conversations between providers and ASHCN could help build competence in self-management by focusing on basic health information needed to make appropriate decisions; understanding the purpose of medications and other treatments and when they need to see their provider; and practicing how to ask their provider about health concerns. Nurturing these competencies aligns with the goal of enhancing health literacy, which refers to individuals’ ability to obtain, process, and understand essential health information necessary for making informed health decisions.

Additional factors that contributed ASHCN’s self-efficacy in meeting with their provider independently were identified, offering a framework for interventions aimed at enhancing self-efficacy while still in pediatric care. Cognitive performance is higher and tends to develop earlier in adolescent females, which may contribute to their higher self-efficacy to meet with their providers alone, as in the current report, through being better able to abstract about future health care concerns (Tomasi & Volkow, 2023). Females of this age may also have more experience meeting alone with their provider to discuss reproductive health issues. Provider interventions should take into consideration the ability for abstract versus concrete thinking when promoting competence in self-management with ASHCN. While not significant in the regression model, the SDT constructs of autonomy and parent support were significantly related to self-efficacy at the bivariate level. As expected, provider autonomy support was a stronger predictor than caregiver autonomy support.

Limitations of this study included recruitment during COVID-19 restrictions, a particularly stressful time in general and a time when the health care landscape made significant changes, for example, increased telehealth appointments, that could impact interpersonal experiences between ASHCN, caregivers, and providers. The SDT construct of competence measured a general health care competence. On one hand, general health care competence likely impacts all aspects of health care engagement; on the other, continued research into specific clinic practices (e.g., meeting alone with one’s provider) may also benefit from specific competence assessment of this task. Strengths of this study include focusing on practical and implementable tasks that support ASHCN, specifically 18-year olds, with four different chronic illnesses, developing self-efficacy toward successful transition to adult-based care.

## Conclusion

The findings of this study show that ASHCN self-efficacy to meet with their pediatric provider alone is associated with provider support for this process, including having previously met with the pediatric provider alone. Meeting with the pediatric provider alone is a core element in HCT planning. It is critical for pediatric providers to convey the importance of meeting alone with the ASHCN to the caregiver and the ASHCN and to implement time alone with the ASHCN as a standard practice. The clinical implications of this could include an earlier focus on talking with their pediatric provider alone especially for males and once started, regular implementation of this practice could improve readiness for transition to adult-based care.
